# Type 2 Diabetes Mellitus with Tight Glucose Control and Poor Pre-Injury Stair Climbing Capacity May Predict Postoperative Delirium: A Secondary Analysis

**DOI:** 10.3390/brainsci12070951

**Published:** 2022-07-20

**Authors:** Kaixi Liu, Yanan Song, Yi Yuan, Zhengqian Li, Xiaoxiao Wang, Wenchao Zhang, Yue Li, Xinning Mi, Dengyang Han, Yulan Rong, Xiangyang Guo, Geng Wang

**Affiliations:** 1 Department of Anesthesiology, Peking University Third Hospital, Beijing 100191, China; kathyliu@pku.edu.cn (K.L.); songyanan@bjmu.edu.cn (Y.S.); zhengqianli@hsc.pku.edu.cn (Z.L.); mzliyue@163.com (Y.L.); mixn0106@163.com (X.M.); handengtom@yahoo.com (D.H.); candyrong@foxmail.com (Y.R.); 2 Department of Anesthesiology, Beijing Jishuitan Hospital, 31 Xinjiekou East Street, Xicheng District, Beijing 100035, China; julietyy@sina.com (Y.Y.); wenchaozhang116@163.com (W.Z.); 3 Research Center of Clinical Epidemiology, Peking University Third Hospital, Beijing 100191, China; wxx910129@163.com

**Keywords:** postoperative delirium, glycemic control, physical performance, hip fracture, type 2 diabetes mellitus

## Abstract

(1) Background: Previous evidence demonstrates that tight glycemic control and good physical function could reduce the risk of delirium. This study aimed to investigate whether the occurrence of postoperative delirium (POD) in older hip fracture surgery patients is associated with preoperative glycemic control factors or pre-injury physical performance. (2) Methods: Three-hundred and nine individuals aged over 65 years and scheduled for hip fracture surgery were included at a single center. Glycemic control factors and pre-injury physical performance were assessed preoperatively. The presence of delirium was assessed using the Confusion Assessment Method on postoperative hospitalization days. Univariate and multivariable logistic regression models and a risk prediction model of POD were established. (3) Results: Among the 309 patients, 52 (16.83%) experienced POD during the hospital stay. The numbers of pre-injury physical performance and type 2 diabetes mellitus (T2DM) patients were significantly different in the POD and non-POD groups. The multivariable model showed that development of delirium was significantly explained by preoperative fasting blood glucose (FBG) (OR 0.804, *p* = 0.004), stair climbing (OR 0.709, *p* = 0.003), T2DM (odds ratio (OR) 3.654, *p* = 0.001), and age-adjusted Charlson comorbidity index (ACCI) (OR 1.270, *p* = 0.038). The area under the receiver operating characteristic curve (AUROC) of the risk prediction model including those covariates was 0.770. (4) Conclusions: More older T2DM patients develop POD after hip fracture surgery than patients without T2DM. A simple assessment of preoperative FBG and pre-injury stair climbing capacity may identify those at high risk for the development of POD. Higher preoperative FBG and good pre-injury stair climbing capacity are protective factors for POD.

## 1. Introduction

Postoperative delirium (POD) is characterized by acute disturbances in consciousness, attention, and cognitive impairment. It is a common complication of surgery, especially in older patients.

According to prior studies, the incidence of POD is 15.7–48.0% in older hip fracture patients [1,2]. It is associated with a longer hospital stay, higher healthcare costs, neurocognitive impairment, and increased mortality [3,4]. Although surgical and anesthetic techniques have improved over time, the occurrence of POD remains inevitable because the underlying pathogenesis has not been fully elucidated [5].

From the perspective of clinical practice, identifying predictors of POD may ultimately lead to an improved understanding of this condition, and facilitate the development of specific interventions. To aid early recognition, a series of risk factors, including age, polypharmacy, electrolyte disorders, and diabetes, have been proposed for patients with hip fractures [6,7,8]. Emerging literature has described the positive roles of glycemic control factors, including glucose variability and glycosylated hemoglobin type A1C (HbA1c) level, in predicting outcomes in older patients. High preoperative HbA1c levels and poor glycemic control were reported to increase the risk of POD following cardiovascular surgery [9,10]. Delirium in the intensive care unit (ICU) was shown to be associated with the occurrence of hypoglycemia, but not with pronounced glucose variability [11]. Stress-induced hyperglycemia (SIH) occurs in acute physiological stress and is associated with increased mortality and morbidity in hospitalized people, having a greater negative impact in non-diabetic patients [12]. Glycemic gap (GG) has also been demonstrated to be useful in assessing the disease severity and prognosis [13]. However, the above evidence was limited to nonsurgical patients or patients undergoing non-orthopedic surgery, and no studies have demonstrated how those preoperative glycemic control factors affect the risk of developing delirium in older patients following hip fracture surgery.

In older individuals, especially patients undergoing osteoarthritic surgery, daily activities are the basis for assessing physical performance. Several studies have suggested that impairments of physical performance, such as disability, immobility, and functional decline, are risk factors for POD [14,15,16]. To date, few studies have explored the role of pre-injury physical function in the development of POD in older hip fracture patients.

We therefore conducted the present secondary analysis to investigate the associations between POD and preoperative glycemic control factors and pre-injury physical performance in older hip fracture patients.

## 2. Materials and Methods

### 2.1. Study Design

This secondary analysis was based on two prospective observational, single-center, cohort studies which were conducted at Beijing Jishuitan Hospital. Those two studies investigated the associations of exosome alpha-synuclein [17] and different anesthesia methods with POD, respectively [18,19]. This secondary analysis was approved by the ethics committee of Peking University Third Hospital (M2020562) and was registered at the Chinese Clinical Trial Registry (ChiCTR2100042702). Written informed consent was obtained from each patient before enrollment.

### 2.2. Participants and Features

Inclusion was described previously [17,18,19], and exclusion criteria were modified to include the psychiatric or mental disorder. The eligibility criteria were aged ≥ 65 years old, ASA physical status classification of I–III, experienced a hip fracture, and was admitted to the orthogeriatric (OG) unit. Exclusion criteria were as follows: preoperative delirium, Parkinson’s disease, all-cause dementia (including Parkinson disease-related dementia, Alzheimer’s disease-related dementia, and Lewy’s body dementia), alcohol dependence, multiple trauma or fractures, acute or chronic infectious diseases, stroke within the prior 6 months, any other central nervous system (CNS) disease, severe deafness or vision problems, linguistic barriers, illiteracy, communication difficulties, psychiatric or mental disorder, postoperative transfer to the ICU, and refusal to be discharged or unexpected discharge.

### 2.3. Preoperative Interview

In the original study, we interviewed all patients preoperatively and collected baseline demographics and preoperative data, including sex, age, body mass index (BMI), history of smoking, alcohol use, education years, preoperative Mini-Mental State Exam (MMSE) [20], Activities of Daily Living (ADL) [21], age-adjusted Charlson comorbidity index (ACCI) [22], ASA physical status classification [23], baseline laboratory examination, and medical history. Other information, including intraoperative and postoperative data, was also collected according to medical records. All the history collection, physical evaluation, and cognitive assessment were conducted by the trained investigators.

### 2.4. Glycemic Control Factors

We collected glycemic control factors, such as preoperative fasting blood glucose (FBG), HbA1c, SIH, GG, perioperative insulin administration, hypoglycemia [24], and duration and complications of type 2 diabetes mellitus (T2DM) (diabetic peripheral neuropathy [25], diabetic peripheral vascular disease [26]). Blood samples were taken from patients after admission and before receiving any clinical treatment, after fasting for 8–12 h to test FBG levels. Hyperglycemia is highly associated with increased morbidity and short-term mortality, whereas HbA1c is regarded as a good indicator of long-term (3 months) glycemic control [27]. SIH is a hyperglycemic response to physiological stress in nondiabetic patients. The diagnostic criteria for SIH were as follows: (1) no previous history of diabetes, (2) admission FBG ≥ 7 mmol/L, and (3) normal HbA1c values [28]. The effect of long-term glycemic control on hyperglycemia can be excluded from the calculation of GG in diabetic patients, and GG could be a simple measurement for glucose variability [28,29,30]. GG was calculated as preoperative FBG minus the HbA1c-derived average glucose level [31]. HbA1c values were used to calculate the average glucose level, using the following equation: 28.7 × HbA1c − 46.7 mg dl^−1^ [31].

### 2.5. Assessment of Pre-Injury Physical Performance

To ensure that the different dimensions and accuracy of patient function were captured, the assessment of physical performance was self-reported by patients and verified with family members. Pre-injury physical performance, including resistance capacity, aerobic capacity, balance, and exercise capacity, was collected. Resistance capacity was assessed by maximum stair climbing capacity, aerobic capacity was assessed by daily walking distance, and balance was assessed by use (or lack of use) of a mobility aid before the injury. The daily walking distance and the maximum stair climbing capacity of each patient were recorded based on their self-reporting and living environment. At the OG unit of Beijing Jishuitan Hospital, exercise capacity was assessed by the trained geriatrician and consisted of three levels (excellent: normal daily activities and more than one physical exercise session per week; ordinary: normal daily activities, no physical exercise per week; poor: limitation of daily activities). Four parameters (walking distance, maximum stair climbing capacity, the use of mobility aid, and exercise capacity) of physical performance were recorded in the medical records of each patient.

### 2.6. Anesthesia and Analgesia

Based on protocols established by the OG unit, all participants received an ultrasound-guided single injection of 30 mL of 0.33% ropivacaine for fascia iliac block immediately after admission. Anticholinergics or sedatives were avoided before anesthesia. The choice of anesthesia method (general anesthesia (GA) or spinal anesthesia (SA)) was based on individual situations and the experience of anesthesiologists and surgeons. The bispectral index (BIS) parameter is used to guide the titration of general anesthesia depth, and the reading ranges of BIS were set between 40–60. Furthermore, all patients received standard perioperative pain management, including a single injection of ultrasound-guided fascia iliac block (30 mL of 0.33% ropivacaine) before anesthesia and intravenous patient-controlled analgesia (100 mg sufentanil and 8 mg ondansetron in 100 mL saline) postoperatively. In addition, intramuscular injection of 50 mg pethidine or oral use of 5 mg oxycodone/325 mg acetaminophen was performed as remedy analgesia as needed [17].

### 2.7. Postoperative Assessment

The Confusion Assessment Method (CAM) in the Chinese language has been proven to have good reliability and validity with use in the Chinese elderly population [32], and was used for the diagnosis of delirium in original studies. The CAM algorithm consists of four clinical criteria: (1) acute onset and fluctuating course; (2) inattention; (3) disorganized thinking; and (4) altered level of consciousness. For delirium to be defined, both the first and the second criteria have to be present, plus either: the third or the fourth criteria present, or both the third and fourth criteria present together [33]. Furthermore, all participants were followed up twice daily over a two-day period after surgery by trained geriatricians.

### 2.8. Statistical Analysis

Statistical analyses were performed using SPSS Statistics 21.0 (IBM, Armonk, NY, USA). Continuous variables are presented as mean ± standard deviation or median with an interquartile range as appropriate, and categorical variables are presented as frequencies. The normality of variables was tested using the Kolmogorov–Smirnov method. Differences between groups were compared using the chi-square test for categorical variables, an independent-samples t-test for continuous variables, or the Mann–Whitney U-test for the non-normal variables. Two-tailed *p* values < 0.05 were considered statistically significant. Associations between the risk factors and POD were investigated using univariate and multivariable logistic regression analyses. Factors identified as significant in univariate analysis (*p* < 0.200) were included in multivariable logistic regression analysis, and stepwise backward logistic regression was used. The prediction models of POD were established. In prediction models, the included covariates were selected based on the evidence from prior investigations (preoperative MMSE, ACCI, T2DM) and the results of multivariable logistic regression (preoperative FBG, stair climbing). We performed linear association test (*p* values > 0.05 indicate linear association is reasonable for continuous prediction) and multicollinearity test (variation inflation factors (VIF) < 10 mean no collinearity with each covariate and acceptable) for all covariates in the model. The OR and 95% confidence interval (CI) were plotted using the “forest plot” packages in CENTOS7 R-4.0.2. The AUROC was plotted using Prism 7.0 (GraphPad, San Diego, CA, USA) to evaluate the specificity and sensitivity of models for predicting POD.

## 3. Results

### 3.1. Baseline Features

**Figure 1:**
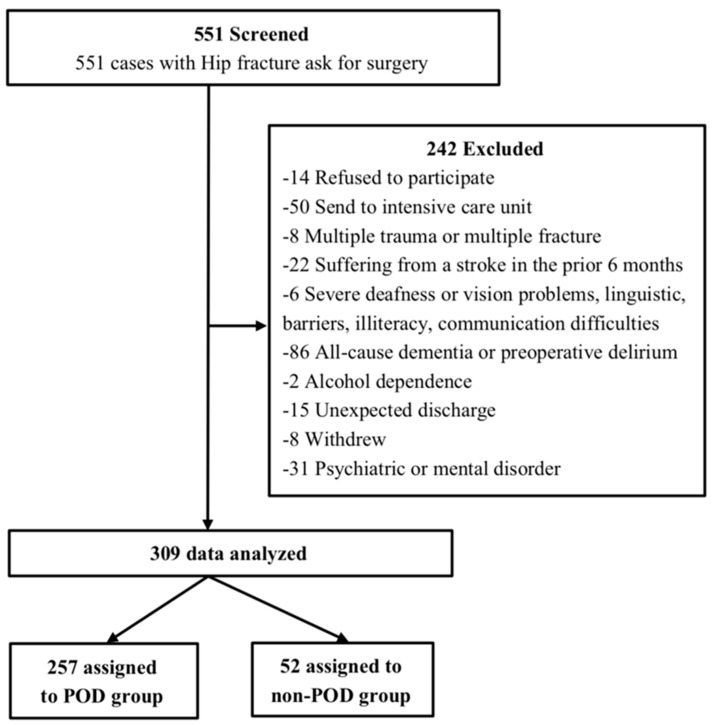
Study flow chart.

Among 551 patients, 309 (183 from cohort 1, 126 from cohort 2) were eligible for inclusion (Figure 1), 16.83% of whom experienced POD during 2 days after surgery. Based on the presence or absence of POD, we divided patients into the POD group and the non-POD group. The mean ages in the POD group and the non-POD group were 79 years and 81 years, respectively. There were 68 male patients (26.46%) in the non-POD group and 16 male patients (30.77%) in the POD group (*p* > 0.05). Baseline demographics and perioperative features between the groups are shown in Table 1.

Patients in the POD group had higher ACCI scores (*p* = 0.003). Except for T2DM (*p* = 0.012), laboratory examination parameters and comorbidities at baseline were not significantly different between the two groups (Table 1). Patients under GA and those who underwent more traumatic surgery (total hip replacement arthroplasty) were more likely to develop POD (*p* = 0.020) (Table 2). Patients in the POD group experienced prolonged hospitalization and had a higher occurrence of postoperative pulmonary infection (*p* = 0.006) (Table 3). No other intra- or postoperative factors were significantly different between the two groups.

### 3.2. Glycemic Control Factors

The median (interquartile range (IQR)) of preoperative FBG for all patients in this study was 7.8 (3.2) mmol/L. The median was 10.1 (5.4) mmol/L for T2DM patients and 7.3 (2.1) mmol/L for patients without T2DM. In T2DM patients, preoperative FBG was significantly lower in the POD group (8.4 (7.5, 10.1) mmol/L) than in the non-POD group (10.6 (8.7, 13.6) mmol/L). No other significant differences in measurements of glycemic control factors were observed between the two groups (Table 4).

### 3.3. Pre-Injury Physical Performance

Four dimensions of the pre-injury physical performance were all significantly limited in the POD group. The walking distance and the maximum number of stairs climbed by patients in the POD group were less than those in the non-POD group (all *p* < 0.001) (Table 5). In addition, there was a higher percentage of patients using mobility aids and ranked lowest in the general exercise capacity category in the POD group (*p* = 0.002, *p* = 0.001).

### 3.4. Univariate Logistic Regression Analysis

Univariate logistic analysis showed that 15 factors, including age, preoperative MMSE, ADL, ACCI, time from injury to surgery, hemoglobin, albumin, hypertension, cardiac interventional therapy history, T2DM, preoperative FBG, exercise capacity, distance walked, use of mobility aid, and stair climbing, were statistically associated with POD in our study (Table 6). There was no significant difference between remaining factors in the univariate logistic analysis.

### 3.5. Multivariable Logistic Regression Analysis

The 15 factors were included in multivariable analysis, and five factors remained using the statistical approach of stepwise backward logistic regression. The results of the multivariable analysis indicated that T2DM and ACCI were independent risk factors for POD in older hip fracture patients. Better stair climbing capacity and higher preoperative FBG were protective factors (Table 7).

### 3.6. Prediction Model for POD

**Figure 2:**
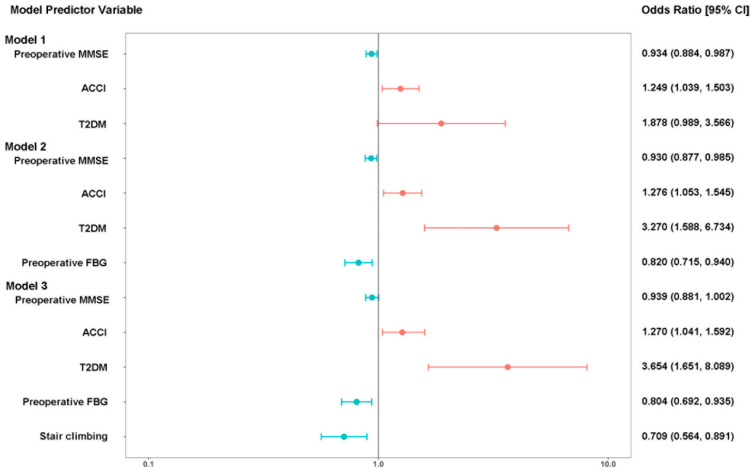
Predictors of POD with OR and 95% CI. MMSE: Mini-Mental State Exam; ACCI: age-adjusted Charlson comorbidity index; T2DM: type 2 diabetes mellitus; FBG, fasting blood glucose.

**Figure 3:**
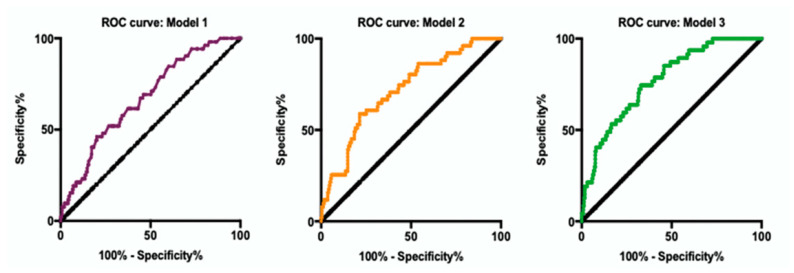
AUROC curve predicted by Model 1, 2, and 3.

The predictors for older hip fracture patients were obtained from multivariable logistic analysis and clinical consensus. The results of linear association test for preoperative MMSE (*p* = 0.065), ACCI (*p* = 0.611), preoperative FBG (*p* = 0.506), and stair climbing (*p* = 0.443) indicated that those continuous predictors are reasonable for prediction. The VIF of five predictors (T2DM (VIF = 1.313), preoperative MMSE (VIF = 1.061), ACCI (VIF = 1.080), preoperative FBG (VIF = 1.233), and stair climbing (VIF = 1.066)), in Model 3 are all less than 10, suggesting that these predictors do not have multicollinearity. We plotted a forest plot (Figure 2) and AUROC (Figure 3) using Models 1–3. In Models 1–3, the predictive value of T2DM increased (OR from 1.878 to 3.270 to 3.654). Based on Model 2, accounting for stair climbing capacity significantly improved the predictive power of POD. The AUROCs of Models 1–3 were 0.672 (95% CI, 0.596 to 0.748), 0.717 (95% CI, 0.642 to 0.792), and 0.770 (95% CI, 0701 to 0.839), respectively.

## 4. Discussion

The primary finding of this study was that older hip fracture patients with T2DM and lower pre-injury physical performance are more likely to develop POD. Although comorbidities (ACCI) continue to be responsible for the increased occurrence of POD [34], the specificity of OG units may eliminate the role of age as a risk factor for POD. Additionally, both preoperative FBG and stair climbing capacity could significantly improve the predictive value of POD.

In this study, we found that T2DM was associated with about 3.654-fold higher odds of POD in the prediction model, including FBG, ACCI, stair climbing capacity, and preoperative MMSE. T2DM is a widely recognized risk factor for the development of delirium after hip fracture surgery [35,36]. However, glycemic control factors were rarely reported in those studies. Many studies demonstrated that both hyperglycemia and hypoglycemia could be risk factors for POD [37,38,39]. Unexpectedly, in T2DM patients, preoperative FBG was higher in the non-POD group (10.6 mmol/L) than in the POD group (8.4 mmol/L). Multivariable logistic regression analysis also indicated that higher preoperative FBG is a protective factor for delirium, especially in T2DM patients. Given that the brain is responsible for over 50% of the total body glucose consumption and depends almost entirely on glucose as an energy source [40], insufficient glucose levels can lead to a lack of energy supply and results in impairment of glucose metabolism, impairment of protein synthesis, ion homeostasis, mitochondrial function, and accelerated lipolysis, ultimately resulting in POD, especially in patients with T2DM who have adaptive mechanisms to chronic hyperglycemia [41,42]. Hypoglycemic episodes in diabetic patients can even predict the development of dementia [43]. In contrast, an adequate supply of glucose may enhance cognitive functioning under stress conditions such as trauma, pain, and anxiety [44]. Saager et al. reported that intensive intraoperative glycemic control (119 ± 18 mg/dL equal to 6.6 ± 1.0 mmol/L) leads to a higher incidence of delirium in patients undergoing cardiac surgery than standard therapy (171 ± 29 mg/dL equal to 9.5 ± 1.6 mmol/L) [45]. Previous studies have also indicated that mild hypoglycemia (3.2 to 3.6 mmol/L) is associated with the impairment of the cognitive performance, brain function and connectivity; and appropriately elevated preoperative glucose levels, rather than tight glycemic control, can be beneficial for the mitigation of POD in older patients [11,46,47].

Besides preoperative FBG, other glycemic control factors (HbA1c, SIH, and GG) were not significantly associated with POD in our cohort. Long-term glycemic control may not play a definitive role in the occurrence of POD, as HbA1c affected less than 10% of the variation in cognitive function [48]. In contrast to our study, Kotfis K et al. reported that elevated preoperative HbA1c level is a risk factor for postcardiac surgery delirium regardless of the diagnosis of diabetes [10]. We speculate that this discrepancy may be because of differences in study populations, types of surgery, and perioperative management patterns. Regarding indicators of glycemic control, SIH and GG are useful parameters for predicting the prognosis of acute myocardial Infarction patients [28,29]. To our knowledge, this is the first trial to include SIH and GG as glycemic control factors for the predicting of POD. Unfortunately, we did not find these factors have predictive value for POD, but they may have significance for measuring patients’ preoperative physical stress status and glycemic control in future studies. SIH is an acute process initiated by injury stress, and stress conditions were similar for nondiabetic hip fracture patients in both groups [49]. In the absence of background glucose level as a context, SIH alone may not be a valid predictor of POD in non-diabetic patients. GG has been used to eliminate the effect of hyperglycemia existing before stress conditions [50]. Patients with high GG have been found to have increased mortality [29,31,51]. Lee M et al. demonstrated that an elevated GG was significantly associated with cognitive impairment after a stroke [52]. However, the paradox of GG has also been reported. Low GG may suggest relative hypoglycemia: a U-shaped relationship between the GG and mortality indicates that relative hypoglycemia is harmful. Unfortunately, none of these studies addressed the association between GG and POD. GG did not show predictive validity and statistical differences in our study. We speculate that it is because under the evolving management of blood glucose at the OG unit, diabetic patients showed good homogeneity in terms of glycemic control and preoperative stress. OG unit represents a new model of comprehensive assessments of older patients by a multidisciplinary team (including orthopedic surgeons, geriatricians, anesthetists, and physiotherapists). The protocols followed at the OG unit address optimal preparation for hip fracture surgery, improved efficiency in perioperative management, and reduced the incidence of delirium following a hip fracture [53].

A strength of our study was that we explored the predictive value of the pre-injury physical performance for POD. In previous studies, assessment of physical function, such as 6 min walk distance and exercise protocols, were helpful for the prediction and improvement of POD, even in frail nonagenarians [54,55,56]. However, neither acute injury nor emergency admission can guarantee preoperative physical assessment and pre-habilitation intervention in elderly patients. Pre-injury daily physical activity is modestly associated with physical performance [57]. Older patients are more likely to be physically inactive, and meeting recommended minimum exercise guidelines appears to be an elusive goal [58]. Therefore, simple daily physical activity may represent a more feasible target [59] and partially represent the physical function. Univariate logistic analysis showed that all of the pre-injury self-reported and informant confirmed physical performance factors were significantly associated with POD. Stair climbing capacity is the most predictive factor among them. Previous studies demonstrated that the stair-climbing test was used as a tool to monitor cardiorespiratory function and represents resistance capacity in fragile older patients [60,61]. Liu, H., et al. reported that changes in daily physical performance were primarily observed in stair climbing capacity and mobility [62].

## 5. Limitations

Several limitations of this study are worth considering. First, this was a single-center study in the OG unit that adopted a specific discharge policy. We only collected data over a 2-day period after surgery, although delirium may occur over longer periods or under other clinical modes. Therefore, the incidence of POD over the entire hospitalization period may have been underestimated. Second, the time points of pain scores collection were not consistent in original studies; intra- and postoperative blood glucose levels were not collected. We therefore cannot exclude the possibility of adverse events caused by increased postoperative pain or perioperative glucose variability. Although the protective effect of higher preoperative FBG levels was explainable, the cutoffs of the protective glycemic level of T2DM (10.25 mmol/L) and non-T2DM patients (6.05 mmol/L) were not consistent. This was an observational study and did not group patients according to glycemic control. The specific upper range of preoperative FBG levels that confers a protective effect in reducing POD was not found. Additionally, there was no background glucose or dynamic glycemic monitoring, so perioperative glucose variability was unable to assess. Third, the-well accepted assessments of physical performance for the elderly, such as FRAIL score, were not performed in the original study. We were unable to use a comprehensive scale to predict the occurrence of POD in this secondary analysis. Finally, we could not improve elderly patients’ physical performance preoperatively in the short term but were still able to use physical performance indicators as predictors of POD. Therefore, we are in great need to have a widely applicable physical performance measurement that is robust across different populations and settings. Although the ability to collect data on physical performance at the OG unit was highly limited, the widespread use of motion tracking systems in smartphones presents a tremendous opportunity to explore more accurate and comprehensive assessments of physical performance in older hip fracture patients.

## 6. Conclusions

Patients with T2DM have a higher risk of developing POD than patients without T2DM. Higher preoperative FBG level and good stair climbing capacity are protective factors for POD; they can be used as potential factors for predicting POD following hip surgery in older patients.

## Figures and Tables

**Table 1:** Baseline features and preoperative characteristics.

Variable	Non-POD (*n* = 257)	POD (*n* = 52)	*p*
**Baseline features**			
**Male (%)**	68 (26.46%)	16 (30.77%)	0.524
**Age (years)**	79 (73, 83)	81 (75, 85)	0.146
**BMI (kg m^−2^)**	23.44 (21.12, 26.20)	23.67 (22.09, 26.19)	0.524
**History of smoking (%)**	49 (19.07%)	11 (21.15%)	0.729
**History of alcohol (%)**	23 (8.95%)	7 (13.46%)	0.316
**Education (years)**	9 (4, 12)	9 (6, 12)	0.628
**Preoperative MMSE (points)**	26 (23, 28)	25.5 (21, 28)	0.065
**Preoperative MMSE < 24**	172 (66.9%)	30 (57.7%)	0.202
**ADL (points)**	6 (6, 6)	6 (4, 6)	0.237
**ACCI (points)**	3 (2, 4)	4 (3, 5)	0.003
**ASA classification (%)**			0.667
** I**	4 (1.65%)	0 (0%)	
** II**	177 (68.78%)	34 (65.38%)	
** III**	76 (29.57%)	18 (34.62%)	
**Baseline laboratory**			
**Hemoglobin (mg L^−1^)**	122 (110, 131)	120 (105, 126)	0.195
**Albumin (g L^−1^)**	41.4 (39.4, 43.4)	40.9 (39.0, 42.1)	0.063
**Abnormal K^+^ (%)**	26 (10.12%)	7 (13.46%)	0.470
**Abnormal Na^+^ (%)**	28 (10.89%)	3 (5.77%)	0.287
**Abnormal Cl^−^ (%)**	57 (22.18%)	9 (17.31%)	0.502
**Abnormal Ca^2+^ (%)**	129 (59.19%)	29 (55.77%)	0.423
**Medical history**			
**T2DM**	69 (26.85%)	23 (44.23%)	0.012
**Hypertension (%)**	146 (56.81%)	36 (69.23%)	0.097
**Coronary heart disease (%)**	58 (22.57%)	13 (25.00%)	0.704
**Kidney failure (%)**	9 (3.50%)	3 (5.77%)	0.705
**Stroke history (%)**	46 (17.90%)	11 (21.15%)	0.608
**Cardiac interventional** **therapy (%)**	6 (2.33%)	4 (7.69%)	0.118

The categorical variables are expressed as n (%). Normal data are given as mean ± SD, whereas non-normal data are expressed as median (25th percentile, 75th percentile). BMI, body mass index; MMSE, Mini-Mental State Exam; ADL, activities of daily living; ACCI, age-adjusted Charlson comorbidity index; T2DM, type 2 diabetes mellitus.

**Table 2:** Intra-operative data.

Variable	Non-POD (*n* = 257)	POD (*n* = 52)	*p*
**Time from injury to operation (hours)**	95 (70, 123)	81 (59, 145)	0.499
**Anesthesia method (%)**			0.001
** SA**	124 (48.25%)	20 (38.46%)	
** GA**	99 (38.52%)	32 (61.54%)	
** SA + GA**	34 (13.23%)	0 (0%)	
**Surgery type (%)**			0.020
** Intramedullary needle**	132 (51.36%)	25 (48.08%)	
** THA**	30 (11.67%)	11 (21.15%)	
** Hemiarthroplasty**	64 (24.90%)	16 (30.77%)	
** Hollow screw fixation**	31 (12.06%)	0 (0%)	
**Anesthesia time (minutes)**	90 (75, 110)	90 (79, 116)	0.454
**Surgery time (minutes)**	60 (40, 80)	60 (38, 90)	0.822

SA, spinal anesthesia; GA, general anesthesia; THA, total hip replacement arthroplasty.

**Table 3:** Postoperative data.

Variable	Non-POD (*n* = 257)	POD (*n* = 52)	*p*
**Postoperative complications (%)**	117 (45.53%)	27 (51.92%)	0.399
**Hypoxemia (%)**	30 (11.67%)	5 (9.62%)	0.669
**Hypokalemia (%)**	14 (5.45%)	6 (11.54%)	0.187
**Pulmonary infection (%)**	11 (4.28%)	8 (15.38%)	0.006
**Deep venous thrombosis (%)**	17 (6.61%)	2 (3.85%)	0.659
**Respiratory failure (%)**	16 (6.23%)	2 (3.85%)	0.731
**Urinary infection (%)**	11 (4.28%)	4 (7.69%)	0.49
**Hypoproteinemia (%)**	10 (3.89%)	3 (5.77%)	0.813
**Hospitalization time (days)**	2 (2, 3)	3 (2, 5)	<0.001

**Table 4:** Glycemic control factors.

Variable	Non-POD (*n* = 257)	POD (*n* = 52)	*p*
**Preoperative FBG (mmol L^−1^)**	7.9 (6.6, 10.1)	7.5 (6.65, 8.9)	0.156
**HbA1c (%)**	5.8 (5.4, 6.5)	6.0 (5.5, 6.7)	0.313
**GG (mg dL^−1^)**	37.9 (10.5, 71.32)	28.5 (8.96, 58.27)	0.245
**Patient without T2DM ^a^**	188 (73.15%)	29 (55.77%)	
** Preoperative FBG** ** (mmol L^−1^) ^a^**	7.4 (6.8, 8.8)	7.1 (5.9, 7.9)	0.084
** HbA1c (%) ^a^**	5.6 (5.3, 5.9)	5.7 (5.32, 6.0)	0.746
** GG (mg dL^−1^) ^a^**	22.7 (8.8, 41.2)	14.4 (6.66, 28.6)	0.112
** SIH (%) ^a^**	90 (47.87%)	14 (48.28%)	0.834
**Patient with T2DM**	69 (26.85%)	23 (44.23%)	
** Preoperative FBG** ** (mmol L^−1^) ^b^**	10.6 (8.7, 13.6)	8.4 (7.5, 10.1)	0.015
** HbA1c (%) ^b^**	6.9 (6.3, 8.0)	6.7 (6.0, 7.5)	0.285
** GG (mg dL^−1^) ^b^**	37.9 (10.5, 71.3)	28.5 (9.0, 58.3)	0.245
** Insulin administration (%) ^b^**	19 (27.54%)	5 (21.74%)	0.677
** Hypoglycemia (%) ^b^**	5 (7.24%)	1 (4.35%)	1.000
**Diabetes duration, years ^b^**	8 (5, 12)	10 (9, 12)	0.119
**DPN (%) ^b^**	15 (21.74%)	3 (13.04%)	0.544
**DPVD (%) ^b^**	4 (5.80%)	3 (13.04%)	0.496

FBG, fasting blood glucose; HbA1c, glycosylated hemoglobin, type A1C; GG, glycemic gap; T2DM, type 2 diabetes mellitus; SIH, stress-induced hyperglycemia; DPN, diabetic peripheral neuropathy; DPVD, diabetic peripheral vascular disease. ^a^ Data were extracted from non-T2DM patients; ^b^ data were extracted from T2DM patients.

**Table 5:** Per-injury self-reported physical performance *.

Variable	Non-POD (*n* = 250)	POD (*n* = 49)	*p*
**Distance walked (meters)**	1000 (500, 2000)	500 (200, 1000)	<0.001
**Stair climbing (floors)**	2 (1, 4)	1 (0, 2)	<0.001
**Use of mobility aid (%)**	61 (23.74%)	23 (44.23%)	0.002
**Exercise capacity (%)**			0.001
** Poor**	25 (10.00%)	12 (24.49%)	
** Ordinary**	95 (38.00%)	24 (48.98%)	
** Good**	130 (52.00%)	13 (26.53%)	

* Physical performance was assessed by a geriatrician in Beijing Jishuitan hospital based on the patient’s general condition.

**Table 6:** Univariate logistic analysis.

Variable	B	SE	Walds	*p*	OR [95% CI]
**Male**	−0.211	0.332	0.405	0.525	0.810 [0.422, 1.552]
**Age, per year**	0.031	0.023	1.820	0.177 ^#^	1.031 [0.986, 1.079]
**BMI, per kg/m^2^**	0.030	0.043	0.489	0.484	1.030 [0.948, 1.120]
**Smoker**	0.130	0.375	0.120	0.729	1.139 [0.546, 2.374]
**Alcohol Drinker**	0.459	0.461	0.995	0.320	1.583 [0.641, 3.909]
**Education, per year**	0.014	0.028	0.479	0.632	1.014 [0.959, 1.072]
**Preoperative MMSE,** **per point**	−0.061	0.027	5.111	0.024 ^#^	0.941 [0.892, 0.992]
**ADL, per score**	−0.192	0.092	4.365	0.037 ^#^	0.825 [0.689, 0.988]
**ACCI, per score**	0.268	0.090	8.902	0.003 ^#^	1.307 [1.096, 1.559]
**ASA classification**	0.232	0.322	0.518	0.472	1.261 [0.671, 2.370]
**Time from injury to** **surgery, per hour**	0.001	0.001	2.414	0.120 ^#^	1.001 [1.000,1.002]
**Anesthesia time, per min**	0.000	0.005	0.009	0.925	1.000 [0.991, 1.010]
**Surgery time, per min**	−0.003	0.005	0.291	0.590	0.997 [0.987, 1.007]
**Hemoglobin, per mg L^−1^**	−0.012	0.008	2.656	0.103 ^#^	0.988 [0.973, 1.003]
**Albumin, per g L^−1^**	−0.087	0.046	3.658	0.056 ^#^	0.917 [0.838, 1.002]
**Hypertension**	0.537	0.326	2.715	0.099 ^#^	1.711 [0.903, 3.239]
**Coronary heart disease**	0.134	0.353	0.144	0.704	1.144 [0.572, 2.286]
**Kidney failure**	0.523	0.685	0.583	0.445	1.687 [0.441, 6.456]
**Stroke history**	0.193	0.377	0.263	0.608	1.213 [0.580, 2.538]
**Cardiac interventional** **therapy**	1.249	0.664	3.532	0.060 ^#^	3.486 [0.948, 12.821]
**T2DM**	0.771	0.313	6.072	0.014 ^#^	2.161 [1.171, 3.988]
**Diabetes duration, per year**	0.031	0.032	0.991	0.319	1.032 [0.970, 1.098]
**DPN**	−0.616	0.684	0.810	0.368	0.540 [0.141, 2.066]
**DPVD**	0.891	0.805	1.224	0.269	2.437 [0.503,11.818]
**Insulin administration, yes**	0.283	0.527	0.288	0.592	1.327 [0.472, 0.731]
**Preoperative FBG,** **per mmol L^−1^**	0.013	0.059	2.438	0.118 ^#^	0.913 [0.814,1.024]
**HbA1c, per%**	0.094	0.329	0.156	0.692	1.139 [0.598, 2.171]
**Exercise capacity**	−0.798	0.222	12.908	0.000 ^#^	0.450 [0.292, 0.696]
**Distance walked, per 100 m**	−0.040	0.015	7.264	0.007 ^#^	0.961 [0.933, 0.989]
**Use of mobility aid, yes**	0.971	0.320	9.217	0.002 ^#^	2.639 [1.411, 4.939]
**Stair climbing, per floor**	−0.386	0.109	12.584	0.000 ^#^	0.680 [0.550, 0.841]

BMI, body mass index; MMSE, Mini-Mental State Exam; ADL, activities of daily living; ACCI, age-adjusted Charlson comorbidity index; T2DM, type 2 diabetes mellitus; DPN, diabetic peripheral neuropathy; DPVD, diabetic peripheral vascular disease; FBG, fasting blood glucose; HbA1c, glycosylated hemoglobin, type A1C; ^#^, *p* < 0.2.

**Table 7:** Multivariable logistic analysis.

Variable	B	SE	Wals	*p*	OR [95% CI]
**Preoperative MMSE, per score**	−0.063	0.330	3.654	0.056	0.939 [0.881, 1.002]
**ACCI, per score**	0.239	0.115	4.319	0.038	1.270 [1.014, 1.592]
**T2DM, yes**	1.296	0.405	10.215	0.001	3.654 [1.651, 8.089]
**Preoperative FBG,** **per mmol L^−1^**	−0.218	0.077	8.076	0.004	0.804 [0.692, 0.935]
**Stair climbing,** **per floor**	−0.344	0.117	8.666	0.003	0.709 [0.564, 0.891]

MMSE, Mini-Mental State Exam; ACCI, age-adjusted Charlson comorbidity index; T2DM, type 2 diabetes mellitus; FBG, fasting blood glucose.

## Data Availability

All data that support the findings of this study are available upon request from the corresponding author.
